# Concurrent Negative Impact of Undernutrition and Heart Failure on Functional and Cognitive Recovery in Hip Fracture Patients

**DOI:** 10.3390/nu15224800

**Published:** 2023-11-16

**Authors:** Shuichi Kamijikkoku, Yoshihiro Yoshimura

**Affiliations:** 1 Department of Cardiology Medicine, Kumamoto Rehabilitation Hospital, Kumamoto 869-1106, Japan; skamijikkoku@kumareha.jp; 2 Center for Sarcopenia and Malnutrition Research, Kumamoto Rehabilitation Hospital, Kumamoto 869-1106, Japan

**Keywords:** frailty, malnutrition, heart failure, rehabilitation nutrition, hip fracture

## Abstract

Evidence on the effects of frailty, undernutrition, and heart failure (HF) on patients with hip fractures is scarce. This retrospective cohort study aimed to examine the effects of undernutrition and HF on outcomes in patients who underwent convalescent rehabilitation after hip fracture. Undernutrition was defined as body mass index (BMI) < 20.0 (Low BMI). Heart failure (HF) was defined as a B-type natriuretic peptide (BNP) > 100 (High BNP). The study outcomes included the Functional Independence Measure motor domain (FIM-motor) and cognitive domain (FIM-cognition) at discharge. To consider the effects of low BMI, high BNP, and the simultaneous presence of both (“low BMI and high BNP”), we used multivariate linear regression analyses to examine whether these were associated with the outcomes. A total of 110 (mean age 87.4 years, 24.8% male) were analyzed. As a result, low BMI (β = −0.088, *p* = 0.027) and high BNP (β = −0.053, *p* = 0.015), each alone, were significantly associated with the FIM motor at discharge, whereas the simultaneous presence of “low BMI and high BNP” was significantly associated with the FIM motor at discharge, while the strength of the association was greater than each association alone (β = −0.152, *p* = 0.010). Further, the simultaneous presence of “low BMI and high BNP” was significantly associated with FIM cognition at discharge (β = −0.109, *p* = 0.014). Comprehensive multidisciplinary management is needed, including preoperative or early postoperative nutritional support and rehabilitation, followed by rehabilitation nutrition care management, in patients with hip fracture.

## 1. Introduction

Frailty and malnutrition are two conditions that are often associated with negative outcomes in older adults. Malnutrition can lead to muscle wasting, weakness, and decreased physical function, which can significantly aggravate the development of frailty [1,2,3]. Frailty, on the other hand, is characterized by a decline in physical function and increased vulnerability to stressors, which can lead to a loss of independence, decreased quality of life, and increased healthcare utilization [4,5,6]. Substantial evidence has shown that frailty is associated with post-operative mortality and adverse events in hip fracture patients [7,8,9]. Factors contributing to this association include reduced physical independence and muscle weakness, prolonged length of hospital stay, and reduced motivation for rehabilitation [10,11]. The association between frailty and malnutrition highlights the importance of comprehensive nutritional assessment and intervention in older adults, including screening for malnutrition and frailty, and interventions that focus on improving nutritional status, physical function, and overall health outcomes [12,13].

Heart failure (HF) is a global pandemic that affects over 26-million adults worldwide and is increasing in prevalence [14,15]. HF represents a collectively experienced chronic phase of cardiac functional impairment that arises as a result of multiple causal factors. Patients afflicted with HF undergo a multitude of symptoms that significantly impact their overall quality of life, including dyspnea, fatigue, reduced exercise capacity, and fluid accumulation [16,17]. While the underlying triggers of HF may vary depending on factors such as gender, age, ethnicity, coexisting medical conditions, and the surrounding environment, it is noteworthy that a considerable number of cases can be prevented [18]. The aging of the population and the emerging pandemic of cardiovascular disease make heart failure an increasing burden in the healthcare system [19]. Further, a meta-analysis of cohort studies with multi-variable adjustment found that patients with HF had a higher risk of hip fracture [20]. Therefore, the management of HF is clinically important in patients with hip fractures.

However, evidence on the effects of frailty, undernutrition, and HF on patients with hip fractures is scarce. Hip fractures are a common injury in older adults that can have significant negative consequences on their health and quality of life [21,22,23]. The effects of frailty, undernutrition, and HF on patients with femoral neck fractures are not well-understood. However, it is important to tackle this problem because hip fractures are associated with a high risk of death and reduced function, and the long-term disability outcomes can be severe. Therefore, this retrospective cohort study aimed to examine the effects of undernutrition and HF on outcomes in patients undergoing convalescent rehabilitation after hip fracture.

## 2. Methods

### 2.1. Participants and Setting

We performed a retrospective cohort study at a post-acute care hospital with a bed capacity of 225, which comprised convalescent rehabilitation wards that had a combined total of 135 beds [24]. This study was grounded in Japan and was executed throughout a span of three years, commencing from July 2021 and concluding in June 2023. All patients who underwent surgery after a proximal femur fracture and were newly admitted to those wards were included in the study. Patients were not included in this study if they declined to provide consent for participation, if they possessed incomplete data, if they necessitated acute care as a result of an exacerbation of a medical condition during the process of rehabilitation, or if they displayed an altered state of consciousness upon admission. Patients were observed until they were discharged.

**Figure 1:**
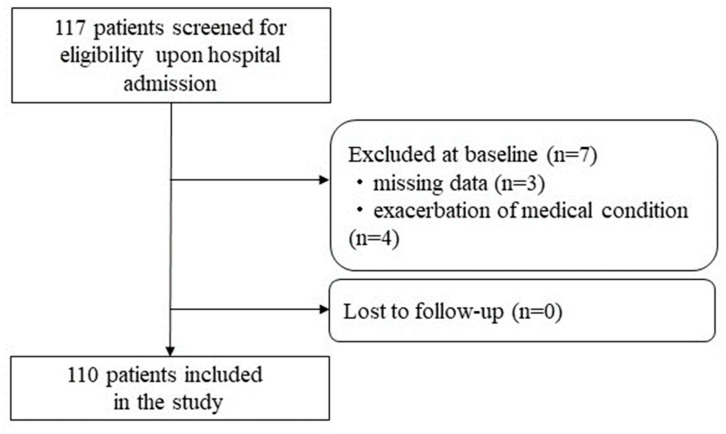
Flowchart of participant screening, inclusion criteria, and follow-up.

A total of 117 femur fracture patients were admitted to the wards for the first time during the study period. Individuals who needed acute care because their medical condition was worsening (*n* = 4) or had missing data (*n* = 3) were not included. Ultimately, 110 patients’ data were incorporated into the analysis (Figure 1).

### 2.2. Data Collection

Patients’ background characteristics, such as age (year), sex, type of femoral fracture, smoking history, fragility fracture history, residential style, comorbidities and pre-existing diseases and conditions, body mass index (BMI, kg/m^2^), activities of daily living, cognitive level, premorbid care burden level [25], serum level of B-type natriuretic peptide (BNP, pg/mL) as a marker of heart failure severity [26], glomerular filtration rate (eGFR, mL/min/1.73 m^2^), and left ventricular ejection fraction (LVEF, %) and E/e′ ratio by echocardiography, were recorded upon admission.

BMI was computed by dividing the weight by the square of the height, while the nurse conducted physical assessments on the day of admission. The premorbid care burden level was assessed through the certification of “required support” and “required long-term care” within Japan’s Long-Term Care Insurance System [25]. This included a total of eight levels: no certification, support needed 1,2, and care needed 1–5. Among the aforementioned tests, blood tests including BNP and eGFR, as well as echocardiography, were routinely conducted on all patients upon admission to determine the intensity, duration, and type of rehabilitation.

### 2.3. Nutrition Assessment

A variety of methods have been reported to diagnose and assess frailty [27,28]. On the other hand, there is an internationally standardized diagnostic criterion, the Global Leadership Initiative on Malnutrition (GLIM) criteria, for the diagnosis of undernutrition [29]. The GLIM criteria recommend the use of physical indicators such as BMI and weight loss in the diagnosis of undernutrition. Low body weight and low BMI are indicators of malnutrition and frailty in older adults. Other indicators of malnutrition include weight loss of more than 5% in the last 6 months, moderate and severe decrease in food intake, and unintended loss in body weight of more than 5% in 3 months [30]. Therefore, while low body weight and low BMI should be used in conjunction with other indicators for a more comprehensive assessment of frailty and undernutrition, they are clinically easy to use and useful in the detection of malnutrition and frailty. In this study, a BMI of less than 20.0 kg/m^2^ was used as an indicator of frailty and undernutrition as “low BMI” [29].

### 2.4. Cardiac Function Assessment

Blood tests and echocardiography were used to measure BNP, LVEF, and E/e′ to estimate cardiac function and the severity of heart failure. BNP is a hormone produced by the heart that helps regulate blood pressure and fluid balance. It is a valuable marker for diagnosing and monitoring heart conditions, particularly heart failure, and plays a crucial role in guiding treatment decisions for affected individuals. A cutoff value of 100 pg/mL for BNP has been used with high accuracy for the diagnosis of acute heart failure, and this value was used as the reference value in this study [31]. E/e′ is the ratio of the E-wave velocity to the e’ velocity assessed by echocardiography. It is used as an important parameter in echocardiography to assess left ventricular filling pressure and diastolic function. The E/e′ ratio provides insights into the relaxation properties of the heart and is particularly valuable in diagnosing and assessing conditions such as diastolic dysfunction and heart failure with preserved ejection fraction. In this study, HF was defined as a BNP of 100 (pg/mL) or greater as “high BNP.” Further, in order to examine the impact on outcomes of simultaneous low nutrition and heart failure, we defined “low BMI and high BNP” as the simultaneous presence of “low BMI and high BNP”.

### 2.5. Outcomes

The study’s outcomes encompassed the evaluation of the functional independence measure (FIM) scores regarding physical function and cognitive level upon patient discharge [32]. The FIM is partitioned into two distinct domains, namely the motor domain (FIM-motor) and the cognitive domain (FIM-cognition). The motor domain encompasses 13 subitems, while the cognitive domain encompasses five subitems. Evaluations of movements are conducted using a seven-point ordinal scale that ranges from complete assistance to complete autonomy. The range of Total FIM scores spanned from 18 to 126, while the range of FIM-motor scores extended from 13 to 91, and the range of FIM-cognition scores extended from 5 to 35. It should be noted that lower scores were indicative of a higher level of dependence. FIM was evaluated within the first postoperative week. In order to mitigate biases, the FIM was assessed by rehabilitation therapists and nurses who possessed substantial expertise and were entirely separate from the individuals responsible for data collection, evaluation, and analysis, as well as the study’s final determinations.

### 2.6. Convalescent Rehabilitation

Depending on the functional skills and impairments of the patient, various disciplines performed a convalescent rehabilitation program that lasted up to three hours per day. These disciplines comprised speech, occupational, physical, and hearing therapy, as well as diet [33], oral [34], and drug management [35]. The facilitation of paralyzed limbs, range of motion, fundamental mobility, walking, resistance, and ADL training were all included in physical therapy [36].

### 2.7. Sample Size Calculation

Data from an earlier study conducted in the same context [37], which revealed that patients’ FIM-motor scores on hospital admission were normally distributed with a standard deviation of 23.4, were used to determine the sample size. In order to reject the null hypothesis with a power of 0.8 and an alpha error of 0.05, a minimum of 31 participants in each group would be needed if the true difference in the mean values between the two groups of low- and high-frequency diet prescriptions issued during hospitalization, with a median frequency cutoff, was 17 [38]. This would support the validity of our findings.

### 2.8. Statistical Analysis

For parametric data, means (SD) were reported; for non-parametric and categorical data, medians (interquartile range; IQR) and counts (%) were reported. Patients were split into three groups (low BMI versus normal BMI, high BNP versus normal BNP, low BMI and high BNP versus other) for the bivariate analysis. The *t*-test, Mann–Whitney U test, and chi–square test were used to compare groups.

The study employed multivariate linear regression analyses to ascertain the independent associations between the baseline variables of low BMI, high BNP, and low BMI and high BNP, as well as FIM motor at discharge and FIM cognition at discharge, respectively. The following covariates were chosen to account for bias: sex, age, length of hospital stay, history of fragility fractures, premorbid care burden level, and baseline FIM-motor and FIM-cognition scores. Based on prior research identifying these variables as clinically significant predictors of rehabilitation outcomes, these factors were chosen [8]. Using the variance inflation factor (VIF), multicollinearity was evaluated: Multicollinearity was absent when the VIF value was between 1 and 10. Statistical significance was defined as *p*-values < 0.05. IBM SPSS version 21 (Armonk, NY, USA) was used for all analyses.

### 2.9. Ethics

The study was approved by the institutional review board of the Kumamoto Rehabilitation Hospital, where it was conducted (approval number: 232–230804). The study was done retrospectively, so we were unable to obtain written informed consent. The study afforded participants the flexibility to discontinue participation at any point through an opt-out process. The 1964 Declaration of Helsinki, its revisions since 1964, and the Ethical Guidelines for Medical and Health Research Involving Human Subjects (Provisional Translation as of March 2015) were followed in conducting the study.

## 3. Results

Patients’ baseline characteristics are presented in Table 1. Mean (SD) age was 87.4 (7.1) years. Twenty five percent of the included patients were male. Recorded fracture types included neck fracture (*n* = 42; 38.5%), trochanteric fracture (*n* = 64; 58.7%), subtrochanteric fracture (*n* = 2; 1.8%), and shaft fracture (*n* = 1; 0.9%). Median (IQR) baseline BMI and serum BNP levels were 21.4 (18.9, 23.6) and 89.9 (50.4, 177.9), respectively. The median (IQR) baseline FIM-motor and FIM-cognition scores were 22 (17, 28) and 20 (13, 30), respectively, suggesting a large proportion of patients were physically dependent.

Table 2 shows the results of the two-group comparison for outcomes. Compared with patients with normal BMI, “low BMI” patients were likely to have lower scores of FIM-motor and FIM-cognition at discharge, respectively. This trend was similar in the two-group comparison of outcomes using “BNP” and “BMI and BNP”.

Table 3 shows the results of multivariate linear regression analyses for FIM-motor at discharge. There was no multicollinearity among the variables, and the same covariates were used in all multivariate analyses for adjustments. As a result, low BMI (β = −0.088, *p* = 0.027) and high BNP (β = −0.053, *p* = 0.015), each alone, were significantly associated with FIM-motor at discharge, whereas the simultaneous presence of “low BMI and high BNP” was significantly and negatively associated with FIM-motor at discharge, while the strength of the association was greater than each association alone (β = −0.152, *p* = 0.010).

The findings of multivariate linear regression analyses for FIM-cognition at discharge are displayed in Table 4. There was no multicollinearity among the variables, and the same covariates were used in all multivariate analyses for adjustments. As a result, low BMI and high BNP, each alone, were not significantly associated with FIM-cognition at discharge, whereas the simultaneous presence of “low BMI and high BNP” was significantly and negatively associated with FIM cognition at discharge (β = −0.109, *p* = 0.014).

## 4. Discussion

We investigated the relationship between functional outcomes, HF, and undernutrition in patients receiving convalescent rehabilitation following a hip fracture in this cohort study. Two new findings are added by our results: (1) Patients with a simultaneous presence of malnutrition and HF had worse ADL improvement; (2) Patients with a simultaneous presence of malnutrition and HF had a worse cognitive level improvement in this setting.

A concurrent presence of undernutrition and HF was negatively associated with ADL recovery, and the strength of the association was greater for undernutrition and HF than for each alone. The novelty of the finding is that it highlights the importance of considering the combined effects of multiple comorbidities on health outcomes in older patients with a hip fracture. Possible mechanisms for the dual burden of undernutrition and HF include the following: undernutrition can cause muscle wasting, weakness, and impaired physical function, thereby exacerbating the negative effects of HF on physical function and overall health [39,40,41,42]. HF, in turn, can lead to decreased cardiac output, reducing the supply of oxygen and nutrients to the muscles and exacerbating muscle loss [43]. The clinical implications of the findings are that clinicians should consider interventions that address both conditions simultaneously, such as nutritional interventions and HF management strategies, to improve physical function and overall health outcomes in hip fracture patients with comorbid undernutrition and HF.

The concurrent presence of undernutrition and HF was negatively associated with cognitive levels recovery. This finding highlights the importance of considering the combined effects of multiple comorbidities on cognitive decline in older adults. The possible mechanism behind the negative association in hip fracture patients is not fully understood. However, some possible mechanisms that could explain this association include: (1) Reduced oxygen and nutrient delivery to the brain due to decreased cardiac output in HF patients, which can exacerbate the negative effects of undernutrition on cognitive function [44,45]; (2) Inflammation and oxidative stress, which are common in both undernutrition and HF, can lead to neuronal damage and cognitive impairment [46]. Indeed, HF patients exhibit abnormal protein metabolism (accelerated catabolism and decreased assimilation) due to chronic inflammation and high oxidative stress [47,48]. Further, chronic inflammation is a risk factor for muscle wasting diseases such as sarcopenia and cachexia in older adults [49,50,51]; (3) The combination of undernutrition and HF may lead to a higher risk of infections, which can further impair cognitive function [52]. Clinical implications include the need for comprehensive nutritional assessment and intervention in hip fracture patients with comorbid undernutrition and HF, as well as HF management strategies to improve cognitive function and overall health outcomes.

Despite the existence of previous studies partially directing our findings in patients with hip fractures, there is a paucity of research showing any association between undernutrition and a higher incidence of heart failure or negative outcomes. For example, there is one observational study of 155 patients that showed an association between undernutrition and the presence of HF and ADL [53], and one study of 172 patients that showed an association between undernutrition and the presence of HF and cognitive levels [54]. However, our findings may represent a novelty showing a stronger negative association between the co-occurrence of malnutrition and HF and improved ADL and cognitive levels than malnutrition alone or HF alone.

The nutritional and cardiac management of patients after a hip fracture is important. Indeed, cardiac and thromboembolic risk constitute the majority of the evidence supporting the perioperative medical risk management of patients undergoing hip fracture repair [55]. However, little is known of the relative clinical importance of other complications, such as undernutrition and cognitive impairment. To prevent the decline in ADL and cognitive level and to help maximize the improvement in outcomes in these patients, comprehensive multidisciplinary management, including nutritional management and rehabilitation before or early after surgery, is necessary [56,57,58]. Furthermore, since older patients after hip fractures are at a higher risk for malnutrition and sarcopenia [37,59], the triad of rehabilitation, nutrition, and oral management should be implemented while managing HF [60,61].

This study had some limitations. First off, the fact that this was a single-center study conducted at a Japanese rehabilitation hospital may have limited how broadly applicable our findings can be. To replicate current findings in a variety of populations, future multicenter studies will be necessary. Second, we were unable to fully account for the impact of confounding factors because the study was retrospective in nature. Third, due to sample-size limitations, the number of patients was biased according to the group being compared. This could affect the reliability of the statistical analysis and interpretation of the results. Future prospective studies should account for these potential confounders.

## 5. Conclusions

The concurrent presence of undernutrition and HF was negatively associated with physical and cognitive recovery in patients with hip fractures. Further, the strength of the association was greater for undernutrition and HF than for each alone. Comprehensive multidisciplinary management is needed, including preoperative or early postoperative nutritional support and rehabilitation, followed by rehabilitation nutrition care management.

## Figures and Tables

**Table 1:** Baseline background of femur fracture patients undergoing rehabilitation.

	Total (*n* = 110)
Age, years	87.4 (7.1)
Sex, male, *n* (%)	27 (24.8)
Fracture type, *n* (%)	
Neck fracture	42 (38.5)
Trochanteric fracture	64 (58.7)
Subtrochanteric fracture	2 (1.8)
Shaft fracture	1 (0.9)
Current smoking, *n* (%)	6 (5.5)
Fragility fracture history, *n* (%)	36 (33.3)
Living at home, *n* (%)	69 (65.1)
Disease/History	
Hypertension	74 (67.9)
Hyperlipidemia	17 (15.6)
Type 2 diabetes	21 (19.3)
Osteoporosis	44 (69.8)
Stroke	18 (16.4)
Orthostatic hypotension	11 (10.1)
Ischemic heart disease	12 (11.0)
Arrhythmia	27 (24.8)
BMI, kg/m^2^	21.4 (18.9, 23.6)
FIM-total	43 (32, 55)
FIM-motor	22 (17, 28)
FIM-cognition	20 (13, 30)
Premorbid care burden level	
None/Support 1/Support 2/Care 1/Care 2/Care 3/Care 4/Care 5	37 (33.9)/5 (4.6)/5 (4.6)/10 (9.2)/21 (19.3)/18 (16.5)/10 (9.2)/3 (2.8)
BNP	89.9 (50.4, 177.0)
LVEF, %	67.0 (63.7, 70.0)
E/e′ ratio	13.8 (11.5, 16.4)
eGFR	59.2 (50.0, 71.8)
Length of stay, days	80 (61, 94)

BMI, body mass index; BNP, B-type natriuretic peptide; eGFR, estimated glomerular filtration rate; FIM, Functional Independence Measure; LVEF, left ventricular ejection fraction. For parametric data, the expression is the mean (standard deviation); for nonparametric data, it is the median and the 25th-to-75th percentiles (interquartile range; IQR); for categorical data, it is numbers (%).

**Table 2:** Univariate analysis between 2 groups based on cutoff values of BMI, BNP, BMI, and BNP for each outcome (FIM exercise, cognition at discharge) in patients with femur fracture.

	Total (*n* = 110)	BMI			BNP			BMI & BNP		
		Normal (*n* = 67)	Low (*n* = 43)	*p*-Value	Normal (*n* = 74)	High (*n* = 36)	*p*-Value	Normal (*n* = 95)	Abnormal ^#^ (*n* = 15)	*p*-Value
FIM-motor at discharge	61 (32, 81)	67 (36, 83)	41 (25, 66)	0.004	63 (37, 82)	41 (24, 67)	0.024	62 (34, 81)	29 (14, 41)	0.001
FIM-cognition at discharge	21 (14, 32)	25 (16, 33)	17 (13, 22)	0.003	23 (15, 33)	17 (13, 24)	0.032	21 (15, 32)	16 (6, 21)	0.013

^#^ Abnormal: Low BMI and high BNP. BMI, body mass index; BNP, B-type natriuretic peptide; FIM, Functional Independence Measure. For parametric data, the expression is the mean (standard deviation); for nonparametric data, it is the median and the 25th-to-75th percentiles (interquartile range; IQR); for categorical data, it is numbers (%).

**Table 3:** Multiple linear regression analysis to examine the association of Low BMI, High BNP, and Low BMI and High BNP to FIM-motor at discharge.

	Low BMI		High BNP		Low BMI and High BNP	
	β	*p*-Value	β	*p*-Value	β	*p*-Value
Age	−0.027	0.652	−0.014	0.823	0.015	0.807
Sex, male	−0.072	0.221	−0.070	0.237	−0.047	0.420
Premorbid care burden ^#^	−0.232	0.001	−0.241	0.001	−0.236	0.001
Fragility fracture history	−0.070	0.221	−0.077	0.182	−0.069	0.217
Length of stay	0.280	0.000	0.275	0.005	0.264	0.011
FIM motor	0.240	0.001	0.220	0.002	0.206	0.002
FIM cognition	0.428	<0.001	0.454	<0.001	0.462	<0.001
Low BMI	−0.088	0.027	-	-	-	-
High BNP	-	-	−0.053	0.015	-	
Low BMI and High BNP	-	-	-	-	−0.192	0.010

^#^ Premorbid care burden: The level of support/care required before injury was treated as an ordinal variable (No certification: 1, Support needed 1: 2, Support needed 2: 3, Care needed 1: 4, Care needed 2: 4, Care needed 3: 6, Care needed 4: 7, Care needed 5: 8). BMI, body mass index; BNP, B-type natriuretic peptide; FIM, Functional Independence Measure.

**Table 4:** Multiple linear regression analysis to examine the association of Low BMI, High BNP, and Low BMI and High BNP to FIM cognition at discharge.

	Low BMI		High BNP		Low BMI and High BNP	
	β	*p*-Value	β	*p*-Value	β	*p*-Value
Age	−0.043	0.328	−0.022	0.638	−0.014	0.762
Sex, male	−0.033	0.448	−0.031	0.472	−0.015	0.727
Premorbid care burden ^#^	−0.103	0.051	−0.110	0.035	−0.106	0.040
Fragility fracture history	−0.051	0.235	−0.053	0.208	−0.051	0.221
Length of stay	0.116	0.007	0.110	0.011	0.104	0.014
FIM motor	0.113	0.026	0.090	0.077	0.088	0.079
FIM cognition	0.743	<0.001	0.766	<0.001	0.771	<0.001
Low BMI	−0.073	0.092	-	-	-	-
High BNP	-	-	−0.083	0.061	-	-
Low BMI and High BNP	-	-	-	-	−0.109	0.014

^#^ Premorbid care burden: The level of support/care required before injury was treated as an ordinal variable (No certification: 1, Support needed 1: 2, Support needed 2: 3, Care needed 1: 4, Care needed 2: 4, Care needed 3: 6, Care needed 4: 7, Care needed 5: 8). BMI, body mass index; BNP, B-type natriuretic peptide; FIM, Functional Independence Measure.

## Data Availability

Due to opt-out restrictions, the data are not publicly available. The sharing of data is not relevant.

## References

[1] Norman K., Haß U., Pirlich M. (2021). Malnutrition in Older Adults-Recent Advances and Remaining Challenges. Nutrients.

[2] Donini L.M., Stephan B.C.M., Rosano A., Molfino A., Poggiogalle E., Lenzi A., Siervo M., Muscaritoli M. (2020). What Are the Risk Factors for Malnutrition in Older-Aged Institutionalized Adults?. Nutrients.

[3] Deutz N.E.P., Ashurst I., Ballesteros M.D., Bear D.E., Cruz-Jentoft A.J., Genton L., Landi F., Laviano A., Norman K., Prado C.M. (2019). The Underappreciated Role of Low Muscle Mass in the Management of Malnutrition. J. Am. Med. Dir. Assoc..

[4] Junius-Walker U., Onder G., Soleymani D., Wiese B., Albaina O., Bernabei R., Marzetti E. (2018). The essence of frailty: A systematic review and qualitative synthesis on frailty concepts and definitions. Eur. J. Intern. Med..

[5] Dent E., Morley J.E., Cruz-Jentoft A.J., Woodhouse L., Rodríguez-Mañas L., Fried L.P., Woo J., Aprahamian I., Sanford A., Lundy J. (2019). Physical Frailty: ICFSR International Clinical Practice Guidelines for Identification and Management. J. Nutr. Health Aging.

[6] Yao A., Zhou S., Cheng J., Kim D.H. (2023). Self-Reported Frailty and Health Care Utilization in Community-Dwelling Middle-Aged and Older Adults in the United States. J. Am. Med. Dir. Assoc..

[7] Yan B., Sun W., Wang W., Wu J., Wang G., Dou Q. (2022). Prognostic significance of frailty in older patients with hip fracture: A systematic review and meta-analysis. Int. Orthop..

[8] Xu B.Y., Yan S., Low L.L., Vasanwala F.F., Low S.G. (2019). Predictors of poor functional outcomes and mortality in patients with hip fracture: A systematic review. BMC Musculoskelet. Disord..

[9] Ma Y., Wang A., Lou Y., Peng D., Jiang Z., Xia T. (2022). Effects of Frailty on Outcomes Following Surgery Among Patients with Hip Fractures: A Systematic Review and Meta-Analysis. Front. Med..

[10] Pizzonia M., Giannotti C., Carmisciano L., Signori A., Rosa G., Santolini F., Caffa I., Montecucco F., Nencioni A., Monacelli F. (2021). Frailty assessment, hip fracture and long-term clinical outcomes in older adults. Eur. J. Clin. Investig..

[11] Song Y., Wu Z., Huo H., Zhao P. (2022). The Impact of Frailty on Adverse Outcomes in Geriatric Hip Fracture Patients: A Systematic Review and Meta-Analysis. Front. Public Health.

[12] Daly R.M., Iuliano S., Fyfe J.J., Scott D., Kirk B., Thompson M.Q., Dent E., Fetterplace K., Wright O.R.L., Lynch G.S. (2022). Screening, Diagnosis and Management of Sarcopenia and Frailty in Hospitalized Older Adults: Recommendations from the Australian and New Zealand Society for Sarcopenia and Frailty Research (ANZSSFR) Expert Working Group. J. Nutr. Health Aging.

[13] Zheng L., Li G., Qiu Y., Wang C., Wang C., Chen L. (2022). Clinical practice guidelines for the prevention and management of frailty: A systematic review. J. Adv. Nurs..

[14] Savarese G., Lund L.H. (2017). Global Public Health Burden of Heart Failure. Card. Fail. Rev..

[15] Savarese G., Becher P.M., Lund L.H., Seferovic P., Rosano G.M.C., Coats A.J.S. (2023). Global burden of heart failure: A comprehensive and updated review of epidemiology. Cardiovasc. Res..

[16] Ziaeian B., Fonarow G.C. (2016). Epidemiology and aetiology of heart failure. Nat. Rev. Cardiol..

[17] Rubio R., Palacios B., Varela L., Fernández R., Correa S.C., Estupiñan M.F., Calvo E., José N., Muñoz M.R., Yun S. (2021). Quality of life and disease experience in patients with heart failure with reduced ejection fraction in Spain: A mixed-methods study. BMJ Open.

[18] Schocken D.D., Benjamin E.J., Fonarow G.C., Krumholz H.M., Levy D., Mensah G.A., Narula J., Shor E.S., Young J.B., Hong Y. (2008). Prevention of Heart Failure. Circulation.

[19] Lam C.S.P., Docherty K.F., Ho J.E., McMurray J.J.V., Myhre P.L., Omland T. (2023). Recent successes in heart failure treatment. Nat. Med..

[20] Liu X.-P., Jian X.-Y., Liang D.-L., Wen J.-X., Wei Y.-H., Wu J.-D., Li Y.-Q. (2022). The Association between Heart Failure and Risk of Fractures: Pool Analysis Comprising 260,410 Participants. Front. Cardiovasc. Med..

[21] Amarilla-Donoso F.J., López-Espuela F., Roncero-Martín R., Leal-Hernandez O., Puerto-Parejo L.M., Aliaga-Vera I., Toribio-Felipe R., Lavado-García J.M. (2020). Quality of life in elderly people after a hip fracture: A prospective study. Health Qual. Life Outcomes.

[22] Ilic I., Ristic B., Stojadinovic I., Ilic M. (2023). Epidemiology of Hip Fractures Due to Falls. Medicina.

[23] Tay E. (2016). Hip fractures in the elderly: Operative versus nonoperative management. Singap. Med. J..

[24] Yoshimura Y., Wakabayashi H., Nagano F., Matsumoto A., Shimazu S., Shiraishi A., Kido Y., Bise T., Hori K., Yoneda K. (2023). Phase angle is associated with sarcopenic obesity in post-stroke patients. Clin. Nutr..

[25] Yamada M., Arai H. (2020). Long-Term Care System in Japan. Ann. Geriatr. Med. Res..

[26] Samad M., Malempati S., Restini C.B.A. (2023). Natriuretic Peptides as Biomarkers: Narrative Review and Considerations in Cardiovascular and Respiratory Dysfunctions. Yale J. Biol. Med..

[27] Fried L.P., Tangen C.M., Walston J., Newman A.B., Hirsch C., Gottdiener J., Seeman T., Tracy R., Kop W.J., Burke G. (2001). Frailty in Older Adults: Evidence for a Phenotype. J. Gerontol. Ser. A Biol. Sci. Med. Sci..

[28] Rockwood K., Mitnitski A. (2007). Frailty in relation to the accumulation of deficits. J. Gerontol. Ser. A Biol. Sci. Med. Sci..

[29] Cederholm T., Jensen G.L., Correia M.I.T.D., Gonzalez M.C., Fukushima R., Higashiguchi T., Baptista G., Barazzoni R., Blaauw R., Coats A.J.S. (2019). GLIM criteria for the diagnosis of malnutrition—A consensus report from the global clinical nutrition community. Clin. Nutr..

[30] Dwyer J.T., Gahche J.J., Weiler M., Arensberg M.B. (2020). Screening Community-Living Older Adults for Protein Energy Malnutrition and Frailty: Update and Next Steps. J. Community Health.

[31] Bozkurt B., Coats A.J., Tsutsui H., Abdelhamid M., Adamopoulos S., Albert N., Anker S.D., Atherton J., Böhm M., Butler J. (2021). Universal Definition and Classification of Heart Failure: A Report of the Heart Failure Society of America, Heart Failure Association of the European Society of Cardiology, Japanese Heart Failure Society and Writing Committee of the Universal Definition of Heart Failure. J. Card. Fail..

[32] Ottenbacher K.J., Hsu Y., Granger C.V., Fiedler R.C. (1996). The reliability of the functional independence measure: A quantitative review. Arch. Phys. Med. Rehabil..

[33] Shimazu S., Yoshimura Y., Kudo M., Nagano F., Bise T., Shiraishi A., Sunahara T. (2021). Frequent and personalized nutritional support leads to improved nutritional status, activities of daily living, and dysphagia after stroke. Nutrition.

[34] Yoshimura Y., Shiraishi A., Tsuji Y., Momosaki R. (2022). Oral Management and the Role of Dental Hygienists in Convalescent Rehabilitation. Prog. Rehabil. Med..

[35] Matsumoto A., Yoshimura Y., Nagano F., Bise T., Kido Y., Shimazu S., Shiraishi A. (2022). Polypharmacy and Its Association with Dysphagia and Malnutrition among Stroke Patients with Sarcopenia. Nutrients.

[36] Nagano F., Yoshimura Y., Matsumoto A., Bise T., Kido Y., Shimazu S., Shiraishi A. (2022). Muscle Strength Gain is Positively Associated with Functional Recovery in Patients with Sarcopenic Obesity after Stroke. J. Stroke Cerebrovasc. Dis..

[37] Yoshimura Y., Wakabayashi H., Bise T., Tanoue M. (2018). Prevalence of sarcopenia and its association with activities of daily living and dysphagia in convalescent rehabilitation ward inpatients. Clin. Nutr..

[38] Beninato M., Gill-Body K.M., Salles S., Stark P.C., Black-Schaffer R.M., Stein J. (2006). Determination of the minimal clinically important difference in the FIM instrument in patients with stroke. Arch. Phys. Med. Rehabil..

[39] Wells J.C., Sawaya A.L., Wibaek R., Mwangome M., Poullas M.S., Yajnik C.S., Demaio A. (2020). The double burden of malnutrition: Aetiological pathways and consequences for health. Lancet.

[40] Rahman A., Jafry S., Jeejeebhoy K., Nagpal A.D., Pisani B., Agarwala R. (2016). Malnutrition and Cachexia in Heart Failure. J. Parenter. Enter. Nutr..

[41] von Haehling S. (2018). Muscle wasting and sarcopenia in heart failure: A brief overview of the current literature. ESC Heart Fail..

[42] Miller M.S., VanBuren P., LeWinter M.M., Lecker S.H., Selby D.E., Palmer B.M., Maughan D.W., Ades P.A., Toth M.J. (2009). Mechanisms Underlying Skeletal Muscle Weakness in Human Heart Failure. Circ. Heart Fail..

[43] Talha K.M., Pandey A., Fudim M., Butler J., Anker S.D., Khan M.S. (2023). Frailty and heart failure: State-of-the-art review. J. Cachexia Sarcopenia Muscle.

[44] Mizrachi E.M., Sitammagari K.K. (2023). Cardiac Syncope. StatPearls [Internet].

[45] Driggin E., Cohen L.P., Gallagher D., Karmally W., Maddox T., Hummel S.L., Carbone S., Maurer M.S. (2022). Nutrition Assessment and Dietary Interventions in Heart Failure. J. Am. Coll. Cardiol..

[46] Hartupee J., Mann D.L. (2017). Neurohormonal activation in heart failure with reduced ejection fraction. Nat. Rev. Cardiol..

[47] Itagaki A., Kakizaki A., Funahashi M., Sato K., Yasuhara K., Ishikawa A. (2019). Impact of heart failure on functional recovery after hip fracture. J. Phys. Ther. Sci..

[48] Inoue T., Maeda K., Nagano A., Shimizu A., Ueshima J., Murotani K., Sato K., Tsubaki A. (2020). Undernutrition, Sarcopenia, and Frailty in Fragility Hip Fracture: Advanced Strategies for Improving Clinical Outcomes. Nutrients.

[49] Ruan G.-T., Xie H.-L., Yuan K.-T., Lin S.-Q., Zhang H.-Y., Liu C.-A., Shi J.-Y., Ge Y.-Z., Song M.-M., Hu C.-L. (2023). Prognostic value of systemic inflammation and for patients with colorectal cancer cachexia. J. Cachexia Sarcopenia Muscle.

[50] Arai H., Maeda K., Wakabayashi H., Naito T., Konishi M., Assantachai P., Auyeung W.T., Chalermsri C., Chen W., Chew J. (2023). Diagnosis and outcomes of cachexia in Asia: Working Consensus Report from the Asian Working Group for Cachexia. J. Cachexia Sarcopenia Muscle.

[51] Yoshimura Y., Bise T., Nagano F., Shimazu S., Shiraishi A., Yamaga M., Koga H. (2018). Systemic Inflammation in the Recovery Stage of Stroke: Its Association with Sarcopenia and Poor Functional Rehabilitation Outcomes. Prog. Rehabil. Med..

[52] Heidenreich P.A., Bozkurt B., Aguilar D., Allen L.A., Byun J.J., Colvin M.M., Deswal A., Drazner M.H., Dunlay S.M., Evers L.R. (2022). 2022 AHA/ACC/HFSA Guideline for the Management of Heart Failure: A Report of the American College of Cardiology/American Heart Association Joint Committee on Clinical Practice Guidelines. Circulation.

[53] Tamamura Y., Matsuura M., Shiba S., Nishikimi T. (2021). Effect of heart failure and malnutrition, alone and in combination, on rehabilitation effectiveness in patients with hip fracture. Clin. Nutr. ESPEN.

[54] Wong A.M., Xu B.Y., Low L.L., Allen J.C., Low S.G. (2021). Impact of malnutrition in surgically repaired hip fracture patients admitted for rehabilitation in a community hospital: A cohort prospective study. Clin. Nutr. ESPEN.

[55] Lawrence V.A., Hilsenbeck S.G., Noveck H., Poses R.M., Carson J.L. (2002). Medical complications and outcomes after hip fracture repair. Arch. Intern. Med..

[56] McDonough C.M., Harris-Hayes M., Kristensen M.T., Overgaard J.A., Herring T.B., Kenny A.M., Mangione K.K. (2021). Physical Therapy Management of Older Adults with Hip Fracture. J. Orthop. Sports Phys. Ther..

[57] Avenell A., Smith T.O., Curtain J.P., Mak J.C., Myint P.K. (2016). Nutritional Supplementation for Hip Fracture Aftercare in Older People. Cochrane Database Syst. Rev..

[58] Takahashi K., Momosaki R., Yasufuku Y., Nakamura N., Maeda K. (2020). Nutritional Therapy in Older Patients with Hip Fractures Undergoing Rehabilitation: A Systematic Review and Meta-Analysis. J. Am. Med. Dir. Assoc..

[59] Yoshimura Y. (2023). Prevention and Treatment of Sarcopenia: Multidisciplinary Approaches in Clinical Practice. Nutrients.

[60] Wakabayashi H. (2023). Triad of rehabilitation, nutrition, and oral management for sarcopenic dysphagia in older people. Geriatr. Gerontol. Int..

[61] Nishioka S., Aragane H., Suzuki N., Yoshimura Y., Fujiwara D., Mori T., Kanehisa Y., Iida Y., Higashi K., Yoshimura-Yokoi Y. (2021). Clinical practice guidelines for rehabilitation nutrition in cerebrovascular disease, hip fracture, cancer, and acute illness: 2020 update. Clin. Nutr. ESPEN.

